# Stress Response and Translation Control in Rotavirus Infection

**DOI:** 10.3390/v8060162

**Published:** 2016-06-07

**Authors:** Susana López, Alfonso Oceguera, Carlos Sandoval-Jaime

**Affiliations:** Departamento de Génetica del Desarrollo y Fisiología Molecular, Instituto de Biotecnología, Universidad Nacional Autónoma de México, Avenida Universidad 2001, Colonia Chamilpa, Cuernavaca, Morelos 62210, Mexico; oca@ibt.unam.mx (A.O.); carlossj@ibt.unam.mx (C.S.-J.)

**Keywords:** rotavirus, antiviral response, stress response, unfolded protein response, protein synthesis, RNA granules, stress granules, processing bodies

## Abstract

The general stress and innate immune responses are closely linked and overlap at many levels. The outcomes of these responses serve to reprogram host expression patterns to prevent viral invasions. In turn, viruses counter attack these cell responses to ensure their replication. The mechanisms by which viruses attempt to control host cell responses are as varied as the number of different virus families. One of the most recurrent strategies used by viruses to control the antiviral response of the cell is to hijack the translation machinery of the host, such that viral proteins are preferentially synthesized, while the expression of the stress and antiviral responses of the cell are blocked at the translation level. Here, we will review how rotaviruses, an important agent of acute severe gastroenteritis in children, overcome the stress responses of the cell to establish a productive infectious cycle.

## 1. Introduction

To successfully replicate, viruses have to cope with the stress and antiviral responses of the host cell. In addition to virus infections, eukaryotic cells encounter a range of physiological and environmental stressful conditions that require adaptive immune responses that induce the coordinated expression of genes affecting cell survival, apoptosis, cell-cycle progression, and differentiation. The endoplasmic reticulum (ER) is an organelle that integrates signals from throughout the cell to orchestrate a coordinated response in these situations. It is the ER where the folding of proteins takes place. Accumulation of misfolded proteins causes ER stress and leads to activation of a coordinated adaptive program called the unfolded protein response (UPR) (reviewed in [1,2]). Also, under stressful conditions cells react with the formation of stress granules (SGs). These are cytoplasmic aggregates of stalled translational pre-initiation complexes that accumulate during stress, increase the amount of Processing Bodies (PBs), which are involved in RNA turnover, and halt the synthesis of most proteins [3,4]. In cells infected with rotavirus there is a shut-off of cell protein synthesis, however, stress granules are not formed, the number of PBs is decreased, and the UPR does not appear to be completely activated. Here we summarize what is currently known about the mechanisms by which rotaviruses evade these responses of the host cell.

## 2. Rotavirus

Rotaviruses are the leading etiologic agents of severe diarrheal disease in infants and young children, being responsible for approximately 200,000 annual deaths globally, and placing a significant economic burden on the global health care system [5,6,7]. Two live attenuated vaccines that protect against the severe form of the disease have been recently licensed, however, the effectiveness of these vaccines is low in developing countries, where rotavirus vaccination is needed most [8,9,10], reinforcing the need to develop alternative approaches to control the infection caused by rotaviruses. Essential for these developments is a basic understanding of the molecular mechanisms by which these viruses interact with their host cell.

As members of the family *Reoviridae*, rotaviruses have a genome made of 11 segments of double-stranded RNA (dsRNA) that is enclosed in a capsid formed by three concentric layers of protein. During cell entry, the viral particle uncoats, losing the two outer surface proteins, VP4 and VP7, and yielding a double-layered particle (DLP) that is transcriptionally active. The viral transcripts direct the synthesis of six structural viral proteins (VPs) and six non-structural proteins (NSPs), and also serve as templates for the synthesis of the complementary strand of the genomic RNA [11]. Early in the infection, once a critical mass of viral proteins is accumulated, large cytoplasmic inclusions termed viroplasms are formed of which the rotavirus NSP2 and NSP5 proteins are essential constituents. The replication of the viral genome, and the assembly of progeny DLPs take place in these structures. The newly synthesized DLPs bud through the ER membrane, modified by the viral transmembrane nonstructural protein NSP4, to yield a particle with an intermediary envelope onto which the outer layer proteins are assembled, the envelope is then lost. The mature viral particles are released by cell lysis or by exocytosis, depending on the cell line [11].

## 3. Protein Synthesis

**Figure 1:**
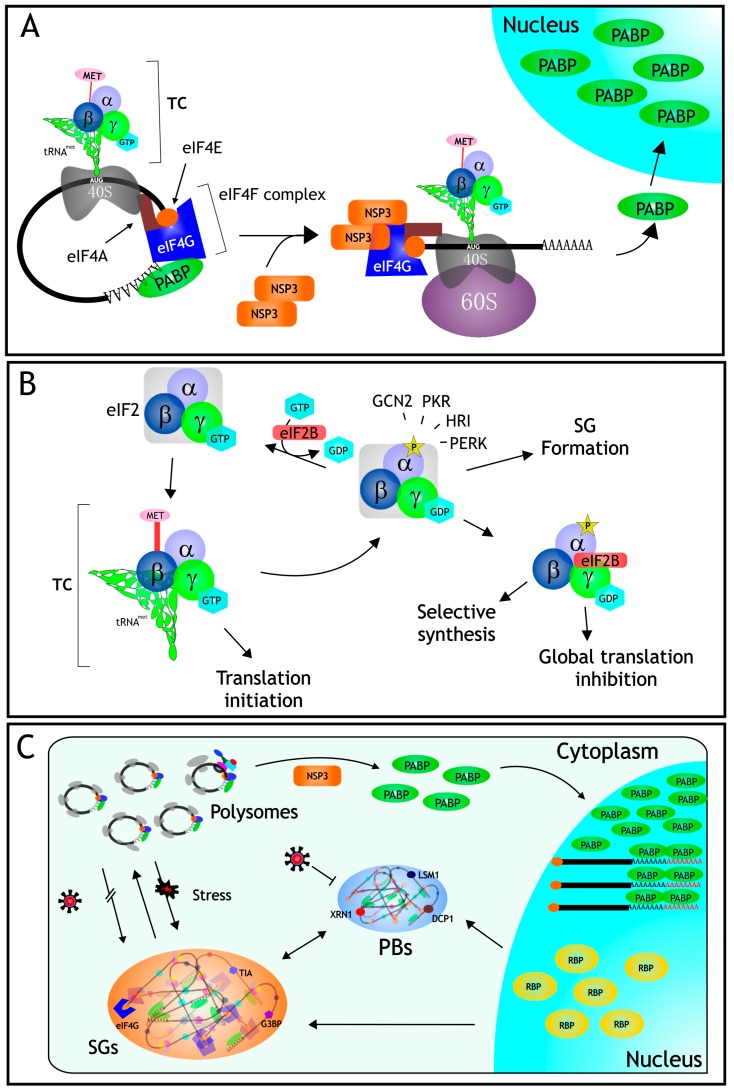
Translation control in rotavirus infected cells. (**A**) During the infection, the nonstructural protein NSP3 interferes with the translation machinery by displacing PABP from its site of binding in eIF4G, and also by indirectly causing the nuclear relocalization of PABP, and the accumulation and hyperadenylation of poly(A)-containing mRNAs (see text); (**B**) eIF2 is an heterotrimer formed by subunits (α, β, and γ); it binds to GTP and tRNA^Met^ forming the ternary complex (TC) which is in charge of loading the initial Met residue during protein translation. During rotavirus infection, the α subunit of eIF2 becomes phosphorylated by PKR, preventing the formation of the TC, and thus causing a global translation inhibition; (**C**) Even under these severe inhibitory translation conditions caused by the infection, the formation of stress granules (SGs) is prevented, and the amount of processing bodies (PBs) decreases, despite the fact that several of the RBPs that assemble these RNA granules exit from the nucleus (see text for details).

As with every process in the cell, the process of protein synthesis is highly regulated, primarily at the level of initiation [12]. In eukaryotic cells, mRNA translation initiation includes the recruitment of mRNAs by the eIF4F complex, and the assembly of the 40S and 60S ribosomal subunits; this process is mediated by the eukaryotic initiation factors (eIFs). The eIF4F complex is constituted by several canonical eIFs which play different roles during translation initiation; eIF4E the cap-binding protein, recognizes the cap structure (mN7GpppG) at the 5′ end of mRNAs; eIF4A is an ATP-dependent RNA helicase that unwinds secondary structures of the mRNAs, and eIF4G, functions as a scaffolding protein on which several eIFs interact. This complex promotes the interaction of the mRNA with the 40S ribosomal subunit [13]. Once the 40S ribosomal subunit is bound to the mRNA, the mRNA is scanned in the 5′–3′ direction, until the first AUG codon surrounded by optimal consensus sequences is found and selected for translation initiation [14] (Figure 1A). A ternary complex (TC) composed of eIF2-GTP-Met-tRNA charges the initiator Met-tRNA to begin translation, and the 60S ribosomal subunit is then joined to form an 80S initiation complex. eIFs are released with the assistance of eIF5, which facilitates the hydrolysis of GTP to GDP carried out by eIF2. The binary complex formed by GDP-eIF2 is recycled by eIF2B that exchanges GDP for GTP, and a new tRNA-Met is loaded to form a ternary complex, ensuing new rounds of initiation [13,15]. The exchange of GDP for GTP in eIF2 is the rate-limiting step in the formation of the ternary complex, and this is regulated by eIF2B (Figure 1B). The two main checkpoints to control translation initiation are the regulation of the activity of eIF2, and the formation of the eIF4F complex. Interestingly, both represent particular targets during the infection by different viruses [16].

## 4. mRNA Translation in Rotavirus Infected Cells

Rotavirus takes over the host translation machinery early in the infection process causing a shut-off of cell protein synthesis, and favoring a robust synthesis of viral proteins such that, by the end of the infection process, most of the newly synthesized proteins in rotavirus-infected cells are viral proteins. To characterize the mechanism by which rotaviruses take over the cellular protein synthesis machinery, several groups have studied the role that viral mRNAs and viral proteins play in this control. In contrast to most cellular mRNAs, rotavirus mRNAs while being capped, are not polyadenylated. Instead, viral mRNAs have a consensus sequence (GACC) at their 3′ end that is conserved in all 11 viral genes [17]. The sizes of the 5′ and 3′ UTRs of viral mRNAs are shorter than those of most cellular mRNAs; the 5′ UTR varies, depending on the specific gene segment, between 9 and 48 bases while the 3′ UTR range from 17 to 182 bases [11].

The role of the UTRs and the 3′ terminal sequence have been studied by different groups using several experimental paradigms. The translation of chimeric reporter genes containing either the 5′ or 3′ rotavirus UTRs, or both [18,19,20,21,22,23], has been analyzed in *in vitro* translation systems in the presence or absence of viral proteins [19,23]. They have also been assayed in different cell lines in which reporter genes have been transfected by either lipofection or electroporation, and their expression has been analyzed in cells infected or not with rotavirus, and in the presence or absence of NSP3. In summary, the results in cells in culture have shown that the GACC-3′ terminal sequence of the viral mRNAs is a translational enhancer that is functional only in infected cells, and depends on the presence of viral proteins, in particular NSP3 (see below), and probably others [20]. Interestingly, in these assays, an enhancement in the translation of the transfected poly(A)-containing reporters has also been observed in infected cells as compared to mock-infected cells [20,22]. The role of the 5′ UTR does not seem to be very relevant for the preferential translation of viral *vs.* cellular mRNAs, but the requirement for the cap structure in the 5′-terminal end to improve the translation of the chimeric reporter genes was clearly established [18,21].

## 5. The Role of the Nonstructural Protein NSP3 during mRNA Translation

**Figure 2:**
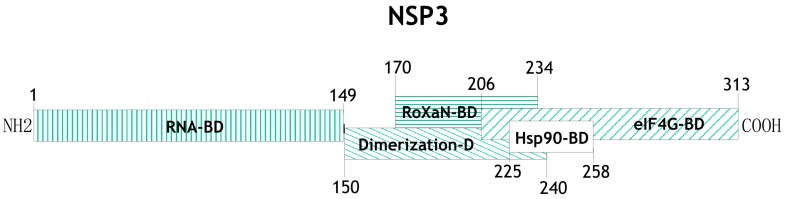
Schematic representation of the different functional domains that have been mapped on NSP3. Numbers indicate the amino acid positions at which each domain starts and ends (BD = binding domain). See text for details and references.

One of the proteins with a relevant participation in the control of cellular mRNA translation and regulation of translation of the viral transcripts is the nonstructural protein NSP3. It has a molecular weight of ~36 kDa, forms homodimers, and several distinct domains have been mapped to different regions of protein (Figure 2). The N-terminal domain (aa residues 1–149) of two NSP3 monomers form an asymmetrical RNA-binding pocket that recognizes the rotaviral mRNA 3′ GACC consensus sequence [17,24,25,26,27], while the C-terminal domain (aa residues 206–313) binds to eIF4G in a region that overlaps with the binding site for the poly(A) binding protein (PABP) [25,26,28]. NSP3 also contains a dimerization domain in the central region of the protein (aa 150–241) that is predicted to form a coiled-coil structure allowing NSP3 dimer formation, and it has been shown that within this region there is a site of interaction with a cellular protein named RoXaN (Rotavirus X protein associated with NSP3); the function of this protein in uninfected as well as in rotavirus-infected cells is still unknown [29]. It has also been shown that the region between amino acid residues 225–258 of NSP3 interacts with Hsp90 and this interaction protects it from proteasomal degradation and helps in the assembly of functionally active dimers of NSP3 [30].

Since NSP3 dimers interact with eIF4G in the same region as PABP does, but with higher affinity [24], it has been proposed that during rotavirus infection NSP3 evicts PABP from eIF4G, impairing the translation of cellular mRNAs, while leading to the enhancement of translation of rotaviral mRNAs [26]. This model was supported by the observation that the synthesis of cellular proteins was severely decreased in cells in which NSP3 was heterologously expressed using a recombinant vaccinia virus [31], and also by *in vitro* translation experiments in which a recombinant NSP3 and its domains were found to stimulate the translation of reporter mRNAs containing the 3′ terminal consensus sequence GACC [23,32]. Interestingly, despite the essential role proposed for NSP3 in infected cells, silencing the expression of this protein by RNA interference (RNAi) experiments showed that, while NSP3 indeed blocked the translation of cellular mRNAs, it was not required for the efficient translation of viral mRNAs. It was also found that the knockdown of NSP3 resulted in an increased production of viral progeny (three-fold more infectious viruses were produced as compared to cells silenced with a control siRNA), and this increase correlated with an increase in the amount of viral mRNA and dsRNA produced under these conditions [33]. Arguments against these assays raise the question as to whether even small undetected amounts of NSP3 are able to initiate the synthesis of viral proteins, at a time in the infection where there is little viral mRNA to compete with the cellular mRNAs [20]. These arguments however, do not explain why more viral progeny is produced under these conditions. Differences in the viral strains used (bovine strain RF or simian strain RRV) and in the cell lines, or in the experimental paradigms used may also account for these discrepancies.

Recently, the translation of reporter genes flanked by either viral or cellular UTRs was compared in cells infected with rotavirus or mock-infected [20,22], or in cells transfected with a plasmid expressing NSP3 [20]. It was found that NSP3 (either produced by the infection, or from a plasmid encoding it) is able to enhance the translation not only of viral RNAs, but also of poly(A)-containing mRNAs. It has been suggested that this viral protein might favor the interaction between the cap-binding protein eIF4E and eIF4G and that it could act as a surrogate PABP during translation initiation; thus, NSP3 *per se* might not be the only factor that inhibits the translation of poly(A)-containing mRNAs in rotavirus-infected cells [20].

In fact, it was recently reported that NSP3 has an additional mechanism to prevent the translation of cellular mRNAs: PABP is a shuttling protein that assists the transport of mRNAs from the nucleus to the cytoplasm, where they become accessible to the translation machinery; during rotavirus infection, PABP becomes accumulated in the nucleus of infected cells [22,34,35,36], and it was found that NSP3, and more precisely the eIF4G-binding domain of this protein, is important for the nuclear localization of PABP [35,36]. NSP3 does not directly interact with PABP [35,36], and silencing the expression of either eIF4GI or eIF4GII, or both, by RNAi in rotavirus-infected cells did not affect the accumulation of PABP in the nucleus, indicating that the displacement of PABP from its binding site on eIF4G is not directly related to its change of localization [22]. Also, it has been proposed that the interaction of NSP3 with RoXaN might be important for the nuclear localization of PABP in infected cells [35]. Interestingly, it was also found that a mutant virus with a partially duplicated NSP3 gene that encodes a protein almost twice as big as the wild type protein, and that has a decreased ability to bind to eIF4G, failed to induce the localization of PABP in the nucleus and to prevent the translation of poly(A)-containing RNAs. Despite this fact, the synthesis of proteins of this mutant and its replication were no different from its wild type counterpart, suggesting that the functions of NSP3 might not be essential for the efficient growth of rotavirus in permissive cell lines [34], and gives support to the observations that silencing the expression of this protein by RNAi has little effect on viral protein synthesis.

The accumulation of PABP in the nucleus of rotavirus infected cells also resulted in the accumulation and hyper-polyadenylation of poly(A)-containing mRNAs [22], suggesting that the shutoff of cell protein synthesis during the infection might be due to a blocking of the nucleo-cytoplasmic transport of polyadenylated mRNAs. This model is supported by the observation that a reporter gene transfected as a plasmid or as a polyadenylated mRNA in rotavirus infected cells had different outcomes; while the translation of the reporter encoded in the plasmid was reduced in infected cells, the translation of mRNAs directly transfected to the cytoplasm was not affected. Also, the nucleo-cytoplasmic distribution of cellular mRNAs was altered in rotavirus-infected cells, since cellular mRNAs accumulated in the cell nucleus as a function of the time post-infection [22].

In rotavirus-infected cells the inhibition of cell protein synthesis is also mediated by a second mechanism since the α subunit of eIF2 becomes phosphorylated early after infection and is maintained in this state throughout the virus replication cycle [36]. When eIF2α is phosphorylated, the exchange of GDP to GTP catalyzed by eIF2B is inhibited, and the eIF2-GDP complex binds with higher affinity to eIF2B preventing its activation. This, in turn, reduces the formation of pre-initiation translation complexes, causing a reduction in global translation [13] (Figure 1B). The permanent phosphorylated status of eIF2α is beneficial for the virus, since under these conditions the viral mRNAs are efficiently translated, while the synthesis of most cellular proteins stops. There are four different kinases that are capable of phosphorylating eIF2α at the same position (Ser-51). These kinases respond to different stress conditions: HRI is activated by heme deficiency, arsenite treatment, or heat shock; PKR is activated by the presence of dsRNA; PERK becomes active when the ER is under stress; and GCN2 is activated under amino acid starvation conditions [37,38]. It has been established that the kinase responsible for the phosphorylation of eIF2α during rotavirus infection is PKR, since in PKR-knockout mouse embryonic fibroblasts (MEFs), or in cells where PKR was knocked-down by RNAi, eIF2α was not phosphorylated upon rotavirus infection. The translation of rotavirus mRNAs is neither affected by the phosphorylation of eIF2α, nor does it depend on it, since viral mRNAs and viral replication are equally effective in cells with or without PKR [39].

Despite the severe translation conditions imposed by virus infection to the host cell, with little ternary complex available to initiate protein synthesis, the viral transcripts are efficiently translated. The molecular mechanisms by which viral protein synthesis takes place have not been identified. However, it has been found that the amount of viral transcripts produced is in the range of 10s of 1000s of molecules per cell, which is only about 10 times short of the amount of the 18S rRNA present in the cell [22]. The huge number of viral mRNAs (of low complexity, since they represent only 11 different transcripts) in a cell where the translation of poly(A)-containing mRNAs is inhibited by at least three different mechanisms (phosphorylation of eIF2α, poly(A)-containing mRNAs sequestered in the nucleus, and eviction of PABP from eIF4G), leaves the translation of viral mRNAs with little competition for the protein synthesis machinery and explains the severe inhibition of host translation caused by rotaviruses.

## 6. RNA Granules

RNA granules are aggregates of RNA and proteins that are present in all eukaryotic cells and play distinct roles in gene regulation, metabolic homeostasis, and response to stress. The two main cytoplasmic RNA granules are Stress Granules (SGs), which are dynamic cytoplasmic aggregates of stalled translational pre-initiation complexes that accumulate during stress, and Processing Bodies (PBs), which contain proteins involved in RNA degradation. The distribution of eukaryotic mRNA between polysomes, SGs, and PBs, in a given cell condition, determines the rate of mRNA translation and degradation, thus influencing gene expression [40,41]. Since SGs and PBs have fundamental roles in the fate of cellular and viral mRNAs, viruses have developed diverse measures to prevent the deleterious effect of these structures during their replicative cycles (reviewed in [42]).

### 6.1. Stress Granules

Besides its effect on protein translation, the phosphorylation of eIF2α is one of the signals that trigger the formation of SGs. It is thought that SGs are sites in which the integrity and composition of mRNAs is monitored, and they are then sent either to translation, degradation, or storage. mRNAs stored in SGs are not degraded, and they are used for rapid reinitiation of translation in cells recovering from stress (reviewed in [4]). These RNA granules contain the small ribosomal subunit, several eIFs, like eIF3, eIF4G, and eIF4E, and PABP and several RNA binding proteins (RBPs) like HuR, G3BP, TIA-1, and TIAR, among others, which are used as markers for these cytoplasmic inclusions (reviewed in [43]). Since the main function of SGs is to arrest protein synthesis until the stressful conditions are resolved, viruses have to deal with these structures in order to ensure the translation of their mRNAs, and various strategies have been developed, including the occupation of these structures during their replication or preventing their formation through different mechanisms [44]. Under stress conditions, the formation of the pre-initiation complex is blocked, either because eIF2α is phosphorylated, the helicase eIF4A is inhibited, or eIF4G is cleaved, and this arrest in translation triggers a signal that causes that several nuclear RBPs, like TIA-1, TIAR, HuR, and G3BP, move to the cytoplasm, where they rapidly aggregate to form the SGs [4], although the precise mechanism involved in SGs formation is still poorly defined.

In rotavirus-infected cells, even though eIF2α is phosphorylated, these granules are not formed despite the fact that TIA-1 exits the nucleus, suggesting that the virus prevents the assembly of these structures, probably by a mechanism that allows the translation of its own mRNAs [36]. Furthermore, it has been shown that rotaviruses are able to prevent the formation of SGs, since these structures are not formed in cells infected with rotavirus when induced to form SGs by treatment with arsenite, a well-characterized SG inducer. The mechanism by which the formation of these structures is prevented during the infection remains to be determined.

### 6.2. Processing Bodies

SGs are closely linked to PBs which contain besides RNA, several enzymes of the RNA decay machinery. In contrast to SGs, PBs are constitutively present in the cytoplasm of cells, although their number and size vary depending on the stress conditions of the cell. There is a variety of proteins present in PBs, which define different subsets of these foci, including proteins involved in mRNA decapping (Dcp1a/Dcp2, and Edc3), decapping enzymes (Ccr4, Caf1, Pan2, and Pan3), exonucleases (XrnI), and RNA binding proteins involved in RNA silencing and in RNA nonsense mediated decay, like DDX3, Rck/p54, GW182, and Lsm1 [45,46,47]. The mechanism of PB formation, similar to that of SGs, is not clear, but it involves the aggregation of the RBPs around mRNAs. It has also been proposed that there is a dynamic exchange of mRNAs between PBs and SGs [48].

It was recently found that different rotavirus strains are able to inhibit or decrease the formation of PBs during the infection. It was reported that the deadenylating Pan3 enzyme was sent to degradation by rotavirus NSP1, while the exonuclease Xrn1 and the decapping enzyme Dcp1 were relocalized from the cytoplasm to the nucleus, and it was also shown that these three PB-components are able to interact, directly or indirectly, with viral RNA [49]. However, the molecular mechanisms by which these proteins were relocalized to the nucleus was not established.

It is interesting to note that, to prevent the formation of either SGs or PBs, it is necessary that the viral particle is replication competent, since it has been shown that transfection of cells with UV-psoralen-inactivated particles do not disrupt the formation of the RNA granules, suggesting that a newly synthesized viral product (either viral RNA or protein) is needed to control the formation of these structures [36,49]. Since several of the components of RNA granules are RBPs, it is tempting to suggest that, during infection, the vast amount of viral transcripts synthesized outcompete the binding of these cellular proteins, functioning as “sponges” that trap RBPs, as it has been recently proposed [50], and preventing their aggregation into RNA granules; this hypothesis however, has not been experimentally verified.

## 7. Unfolded Protein Response

**Figure 3:**
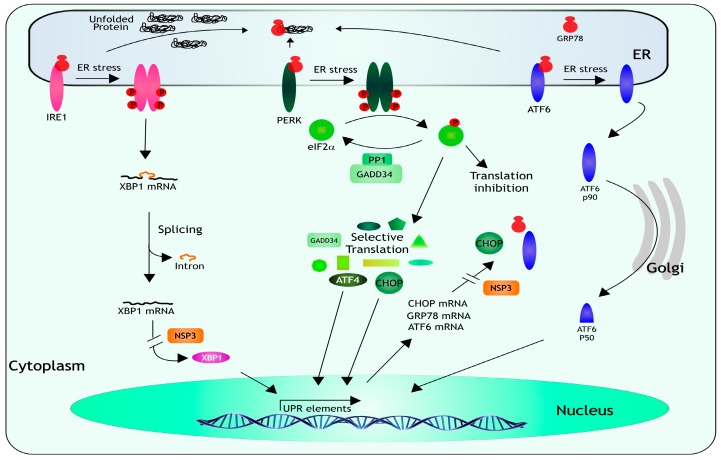
The unfolded protein response. When misfolded proteins accumulate in the ER lumen, GRP78 releases three ER-resident transmembrane proteins, which act as stress sensors: IRE1, PERK, and ATF6. IRE1 has an endonuclease activity that upon dimerization and autophosphorylation mediates the removal of an intron from XBP1 mRNA. The spliced XBP1 encodes a factor that triggers the transcription of genes encoding proteins involved in ER stress-associated protein degradation. Upon activation, PERK phosphorylates eIF2α, resulting in a global inhibition of protein synthesis, and in the selective translation of ATF4 and CHOP, which activate the transcription of several UPR-responsive genes encoding proteins that mitigate the ER-stress, like GADD34, a protein that interacts with PP1 to dephosphorylate eIF2α, to recover protein synthesis.

Besides viral infections, cells have to deal with a range of physiological and environmental stressful conditions that require the coordinated expression of stress-response genes. The ER is in charge of integrating these signals to implement a coordinated response, it is in this cellular organelle where the folding and post-translation modification of proteins takes place; an excess of misfolded proteins causes ER stress and induces the activation of a response named the unfolded protein response (UPR) [1,2] (Figure 3). The main function of the UPR is to decrease the demand on the protein-folding machinery, if the ER stress is not relieved, the activation of apoptotic pathways and cell death take place [1,2]. When misfolded proteins accumulate in the lumen of the ER, three ER-resident transmembrane proteins, which act as stress sensors, are activated: the PKR-like ER kinase (PERK), the activating transcription factor 6 (ATF6), and the inositol-requiring enzyme-1 (IRE1). Under normal conditions, the luminal domain of each sensor is bound to the ER-chaperone GRP78, when UPR is triggered, GRP78 is titered away by the misfolded proteins and releases the sensors allowing their activation. Activation of PERK and IRE1 require dimerization and autophosphorylation, while released ATF6 relocalizes to the Golgi where it is cleaved and activated. When PERK dimerizes, it becomes phosphorylated and then phosphorylates eIF2α, resulting in a downregulation of general protein synthesis, and in the selective translation of ATF4 and CHOP, which in turn activate the transcription of several UPR-responsive genes encoding proteins that ameliorate the ER-stress. Both ATF4 and CHOP induce the transcription of the gene that encodes GADD34, a protein that associates with protein phosphatase 1 (PP1) that dephosphorylates eIF2α, resulting in a negative feedback loop that recovers protein synthesis and permits the translation of the stress-induced transcripts. IRE1 has an RNAse domain that upon dimerization and autophosphorylation mediates the removal of an intron from XBP1 mRNA. The spliced form of XBP1 encodes a factor that activates the transcription of genes encoding proteins involved in ER stress-associated protein degradation. Finally, when GRP78 releases ATF6, it migrates to the Golgi where it is cleaved; p50 one of the cleaved products, is a transcription factor that promotes the transcription of chaperone genes (reviewed in [51,52,53]).

The characterization of the three branches of UPR in rotavirus-infected cells showed that, during the infection, the mRNA of XBP1 is spliced by IRE1, ATF6 is relocalized to the cell nucleus, PERK and eIF2α are phosphorylated, and the transcription of GRP78 and CHOP mRNAs is induced, indicating that the UPR is activated in infected cells [54,55]. Nevertheless, the induction of this cellular response is modulated by rotavirus since GRP78, GRP94, and other proteins involved in the UPR, including PERK, CHOP, and GADD34, are relocalized to/or near viroplasms [55], and the translation of several transcripts induced during the UPR, in particular, the mRNAs for GRP78, XBP1, and CHOP is suppressed by NSP3 [54]. Thus, even though the cell triggers the UPR to confront rotavirus infection, the virus has developed diverse strategies to subvert this coordinated host response to successfully replicate.

When ATF6 is released it migrates to the Golgi where it is cleaved, p50 one of the cleavage products is a transcription factor that promotes the transcription of chaperone genes (reviewed in [51,52,53]). The main function of this coordinated program is to eliminate the misfolded proteins in the ER by increasing the expression of chaperone proteins and degradation factors, and by decreasing the rate of overall protein synthesis to reduce incoming protein traffic into the ER [56]. NSP3 produced in rotavirus-infected cells prevents the translation of spliced XBP1, and newly synthesized CHOP, GRP78, and ATF6 mRNAs, modulating the UPR response [54].

## 8. Concluding Remarks

It seems clear that the regulation of gene expression in cells infected with rotavirus is a multifactorial process. Early during the infection process, the host translation machinery is inhibited since eIF2α becomes phosphorylated by PKR, and the nonstructural protein NSP3 displaces the interaction between PABP and eIF4G; under these conditions, the viral mRNAs are preferentially translated. As the infection proceeds, PABP is relocalized to the cell nucleus by an unknown mechanism, and the newly transcribed cellular mRNAs become hyperadenylated and accumulate in the cell nucleus, unable to reach the cytoplasm and to be translated. At the same time, viral mRNAs accumulate at high rates in the cell cytoplasm with their translation being favored over that of cellular mRNAs. By preventing the translation of cellular genes, the virus controls the activation of the antiviral and UPR responses of the host. Also, during the infection, rotavirus is capable of manipulating the formation of RNA granules, which are deleterious for the viral replication cycle, since the formation of SGs is prevented, and the amount of PBs is decreased.

There are still many questions that need to be answered to fully understand how rotaviruses establish a productive cycle in infected cells. Some of these questions are:

- How can viral and cellular mRNAs be efficiently translated although eIF2α is phosphorylated?
- Does NSP3 participate in the relocalization of PABP to the nucleus?
- Are there additional cellular proteins that interact with NSP3?
- Why does the knockdown of NSP3 result in an increased viral progeny?
- What is the cellular function of RoXaN?
- Which viral proteins are involved in preventing the formation of SGs and PBs?
- What is the function of the UPR proteins found in viroplasms?
- Which is the consequence of suppressing the UPR in rotavirus-infected cells?

## Abbreviations

The following abbreviations are used in this manuscript:

ATF4: Activating Transcription Factor 4
ATF6: Activating Transcription Factor 6
CHOP: C/EBP Homologous Protein (CCAAT-Enhancer-Binding Protein Homologous Proteins)
DCP1: Decapping Protein 1
eIF2B: Eukaryotic Initiation Factor 2B
eIF2α: Eukaryotic Initiation Factor 2 Alpha Subunit
eIF4G: Eukaryotic Initiation Factor 4G
ER: Endoplasmic Reticulum
G3BP: GTPase Activating Protein (SH3 Domain) Binding Protein 1
GADD34: Growth Arrest and DNA Damage-Inducible Protein
GCN2: General Control Nonderepressible 2
GRP78: 78 kDa Glucose-Regulated Protein
HRI: Heme-Regulated Kinase
IRE1: Inositol-Requiring Enzyme-1
LSM1: U6 snRNA-Associated Sm-Like Protein
PABP: Poli(A)-Binding Protein
PBs: Processing Bodies
PERK: Protein Kinase-R-Like ER kinase
PKR: Protein Kinase-R
PP1: Protein Phosphatase 1
RBP: RNA Binding Protein
RoXaN: Rotavirus X Protein Associated with NSP3
SGs: Stress Granules
TC: Ternary Complex
TIA: T-Cell intracellular Antigen 1
UPR: Unfolded Protein Response
UTR: Untranslated Region
XBP1: X-Box Binding Protein 1
XRN1: 5’–3’ Exoribonuclease 1
